# Microstructure and Shear Behaviour of Sn-3.0Ag-0.5Cu Composite Solder Pastes Enhanced by Epoxy Resin

**DOI:** 10.3390/polym14235303

**Published:** 2022-12-04

**Authors:** Peng Zhang, Songbai Xue, Lu Liu, Jie Wu, Qingcheng Luo, Jianhao Wang

**Affiliations:** 1 College of Materials Science and Technology, Nanjing University of Aeronautics and Astronautics, Nanjing 210016, China; 2 Joining and Welding Research Institute, Osaka University, Ibaraki, Osaka 565-0047, Japan

**Keywords:** Sn-3.0Ag-0.5Cu composite solder paste, epoxy resin, spreading performance, microstructure analysis, shear behaviour, fracture morphology

## Abstract

With the rapid development of microelectronics packaging technology, the demand for high-performance packaging materials has further increased. This paper developed novel epoxy-containing Sn-3.0Ag-0.5Cu (SAC305-ER) composite solder pastes, and the effects of epoxy resin on their spreading performance, microstructure, and shear behaviour were analysed. The research results showed that with the addition of epoxy resin, SAC305 solder pastes exhibited exceptional spreadability on Cu substrates, which could be attributed to the reduction in the viscosity and the surface tension of the composite solder pastes. With the addition of epoxy resin, the solder matrix microstructure and interfacial morphology of SAC305-ER composite solder joints remained unchanged. However, continuous resin protective layers were observed on the surface of SAC305-ER composite solder joints after the reflow process. The shear properties of the composite solder joints were enhanced by the extra mechanical bonding effect provided by resin layers. When the epoxy resin content was 8 wt%, the shear forces of SAC305-ER composite solder joints reached the maximum value. Fracture analysis indicated that cracked epoxy resin was observed on the surface of SAC305-ER composite solder joints, indicating that the epoxy resin also underwent obvious deformation in the shear test.

## 1. Introduction

The arrival of the big data era has promoted the broad application of the Internet of Things (IoT) and artificial intelligence (AI). Traditional two-dimensional integrated circuits (2D ICs), limited by Moore’s law, no longer meet the increasing requirement of mobile devices for higher and higher density of electronic components, such as transistors and resistors [1]. Therefore, three-dimensional integrated circuits (3D ICs), consisting of vertically stacked chips, were developed to achieve high performance, miniaturisation, and multifunction of electronic components [2].

With the wide application of 3D ICs, the corresponding packaging technologies developed rapidly, requiring interconnected packaging materials with better solderability and mechanical properties. However, due to the implementation of RoHS 2.0 and WEEE directives, traditional Sn-Pb solder alloys with ideal comprehensive properties are only allowed to be used in some particular fields due to their intrinsic toxicity [3,4]. On this basis, various lead-free solders, including Sn-Ag-Cu, Sn-Cu, Sn-Zn, and Sn-Bi, have been gradually developed and are expected to be potential substitutes for Sn-Pb solders. To make up for the lack of wettability and reliability of lead-free Sn-based solders, two main approaches are taken; one is adding various alloying elements (such as Ga, In, Bi), and the other is the addition of nano or micro particles (such as Ni, Ag, TiO_2_) with high surface energy [5]. In particular, composite lead-free Sn-based solders bearing carbon-based nanomaterials, such as CNTs [6,7] and GNSs [8,9], have attracted more and more attention due to their significantly enhanced mechanical properties and thermal stability. Nevertheless, considering material costs or manufacturing complexity, these solder alloys are still in the academic stage and are rarely used in electronic packaging industries [10].

Epoxy resin is widely used in micro-electronic packaging due to its high strength, corrosion resistance, and sound insulation performance [11]. In recent years, it was found that adding epoxy resin into lead-free solder pastes can notably improve the shear property and thermal reliability of the joints, and thus, epoxy-containing composite solder pastes have become a research hotspot. The cured epoxy resin after the reflow process can adhere to the joint surface and form a composite structure to provide extra mechanical support. Seung-Boo Jung et al. [12,13,14] conducted much research on the mechanical properties and reliability of epoxy-containing Sn-Bi solder paste. It was found that after reflow soldering, the cured epoxy provided an extra bonding effect with the substrate, which increased the shear strength and anti-drop performance of the Sn-Bi epoxy solder joint [12,13]. It was found that during aging, Sn-58Bi epoxy solder joints had a lower IMC (intermetallic compound) growth rate and better mechanical properties than Sn-58Bi solder joints due to the blocking effect of epoxy [14]. Jae Pil Jung et al. [15] evaluated the reliability revolution of epoxy-embedded Sn-3.0Ag-0.5Cu solder pastes during thermal cycling, and found that the epoxy layer could release thermal stress and reduce the Cu-Sn IMC growth. Our group also developed novel Sn-Bi epoxy solder pastes and investigated the influence of epoxy resin content on the solderability and mechanical properties of the composite solder paste [16]. The research result indicated that the appropriate addition of epoxy resin could notably improve the spreadability and shear properties of Sn-Bi solder pastes. Moreover, the thermal reliability and drop characteristics of Sn-Bi epoxy solder joints were enhanced, which could mainly benefit from the mechanical refinement of epoxy resin [17,18].

At present, the research on the mechanical properties and reliability of Sn-58Bi epoxy solder paste has made significant progress. However, there is little research on the effect of adding epoxy resin on the property and reliability of Sn-Ag-Cu solder pastes that are widely applied in reflow soldering due to their desirable thermo-mechanical properties and satisfactory solder performance [19,20,21]. In particular, the effects of epoxy resin content on the spreadability, microstructure, and properties of the solder paste have yet to be systematically studied. Therefore, novel Sn-3.0Ag-0.5Cu (SAC305) composite solder pastes reinforced by epoxy resin were developed in this study. The spreading performance of the SAC305 composite solder pastes was evaluated by a spreading test, and the influence mechanism of epoxy resin and its content on the microstructure and shear behaviour of the corresponding composite solder joints was further investigated.

## 2. Materials and Methods

### 2.1. Sample Preparation

The epoxy-containing Sn-3.0Ag-0.5Cu (SAC305-ER) composite solder paste was prepared by no-clean flux, epoxy resin system, and SAC305 solder power with particle size of 25~45 μm. The epoxy resin system was prepared by mixing E51 thermosetting epoxy resin (Nantong Xingchen Synthetic Material Company Limited, Nantong, China), acid anhydride curing agent, and tertiary amines accelerator (Guangzhou Zhonggao Chemical Company Limited, Guangzhou, China) in proportion. Different from resin-reinforced Sn-Bi solder pastes, in this study, a high-temperature anhydride-type curing agent was selected to match the resin curing temperature with the reflow temperature of the Sn-Ag-Cu solder paste. The accelerator was added to significantly accelerate the curing process. The plain SAC305 solder paste was made only with SAC305 solder power and no-clean flux and was used as control. SAC305-ER composite solder pastes were prepared by adding x (2~12 wt%) epoxy resin into the plain SAC305 solder pastes and recorded as SAC305-xER composite solder pastes.

### 2.2. Spreading Test

The spreading performance of plain SAC305 and SAC305-ER composite solder pastes was evaluated using pure Cu plates as substrates with dimensions of 30 mm × 30 mm × 1 mm. Before the spreading test, these Cu plates were cleaned with 10% dilute sulfuric acid to remove surface oxides and then washed with absolute ethanol. For each specimen, 0.2 ± 0.01 g solder paste was placed on the centre of Cu plates and then reflowed at 250 °C for 3 min using a heating stage. After the spreading test, the spreading area and wetting angle were measured for each parameter, and 5 specimens were prepared repeatedly to calculate the average value.

### 2.3. Shear Test

**Figure 1:**
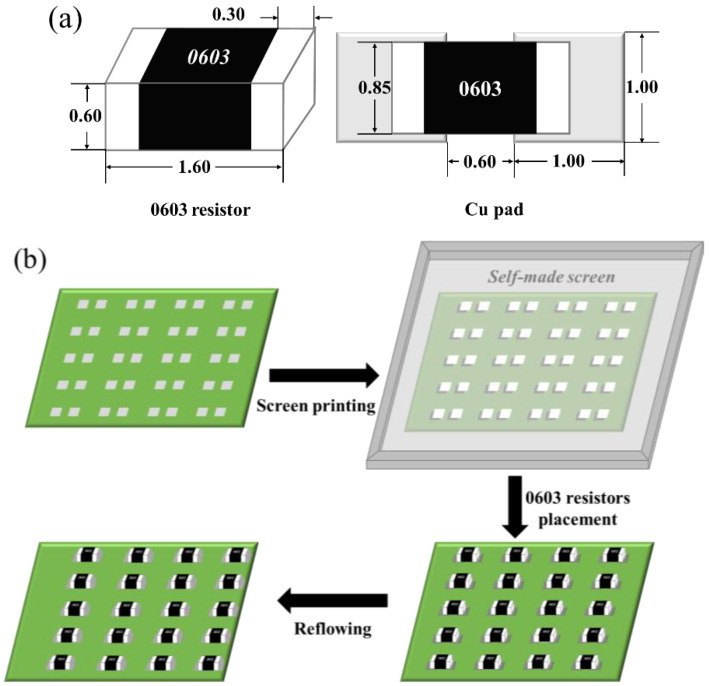
Schematic diagrams of (**a**) dimensions of the 0603 chip resistor and Cu pad (unit: mm) and (**b**) solder joints’ preparation.

The plain SAC305 and SAC305-ER composite solder pastes were printed to the OSP surface finished Cu pads adhered on a printed circuit board (PCB) by the stencil printing method. Then, the 0603 chip resistors were attached to the appropriate position by manual placement. The whole substrate was reflowed with the peak temperature of 250 °C and the total reflow time of 5 min. The packaging dimensions and the schematic of the solder joints’ preparation are shown in Figure 1.

According to Japanese Industrial Standard JIS Z3198-7, the shear test was performed using a micro-joint strength tester (STR1000, Rhesca, Tokyo, Japan), and the speed of the shearing working end was 2 mm/min. After the shear test, shear fracture morphologies of the solder joints were observed by a scanning electron microscope (SEM, SIGMA, Zeiss, Oberkochen, Germany) equipped with energy dispersive X-ray spectrum (EDS, Nano XFlash Detector 5010, Bruker, Billerica, MA, USA).

### 2.4. Microstructure Observation

To observe the cross-sectional microstructure of the solder joints, the specimens were sliced after reflow soldering and ground with sandpaper. After that, these specimens were polished with 0.25 μm diamond paste and etched with alcohol solution containing 4% nitric acid to make phases observable. Microstructure analysis was conducted by SEM and EDS analysis.

## 3. Results and Discussion

### 3.1. Spreading Performance

**Figure 2:**
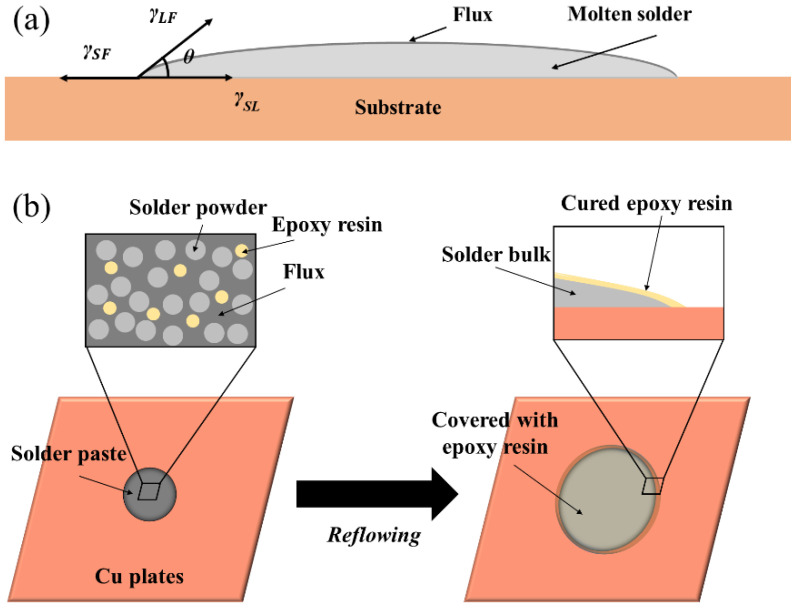
Schematic diagram of (**a**) wetting process of the molten solder on the substrate and (**b**) spreading process of SAC305-ER solder pastes on Cu plate.

At the initial stage of soldering, molten solder first wets and spreads on the surface of the substrate, thereby providing a metallurgical bonding between the solder and the substrate. Therefore, the wetting and spreading performance of the solder are the decisive factors for the formation of solder joints with favourable mechanical properties and reliability. Typically, the larger the spreading area of the solder on the substrates, the better the spreading performance. The wettability of the solder is usually characterised by the contact angle (wetting angle) of the molten solder on the substrate in the equilibrium state. It can be determined by Young’s equation [22] shown below:

(1) $$\cos\theta =\frac{\gamma_{\mathrm{SF}}-\gamma_{\mathrm{SL}}}{\gamma_{\mathrm{LF}}}$$

where *θ* is the contact angle. *γ_SF_*, *γ_SL_*, and *γ_LF_* represent the substrate/flux interface, the substrate/molten solder interface, and the molten solder/flux interface, respectively, as shown in Figure 2a. A smaller contact angle means better wetting of the solder on the substrate.

In this study, a spreading test was conducted to evaluate the proportion of epoxy resin on the spreading performance of SAC305-ER composite solder pastes. During reflowing, the first reaction is the volatilisation of the partial flux and the interaction between the flux and Cu plate to remove the oxides. Subsequently, the solder power was heated and melted while the low-density epoxy resin floated onto the surface of the molten solder alloy and gradually cured. After solidification, the surface of spreading samples of SAC305-ER composite solder pastes was covered with a light yellow epoxy resin layer; the schematic diagram is shown in Figure 2b.

**Figure 3:**
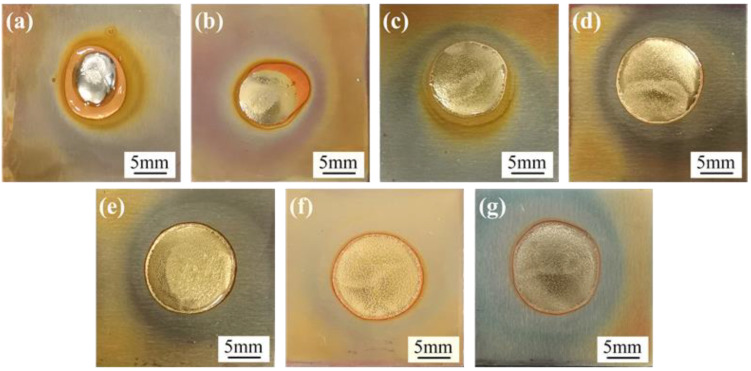
Spreading samples of plain SAC305 and SAC305-xER solder pastes on Cu plates: (**a**) plain SAC305, (**b**) x = 2 wt%, (**c**) x = 4 wt%, (**d**) x = 6 wt%, (**e**) x = 8 wt%, (**f**) x = 10 wt%, (**g**) x = 12 wt%.

**Figure 4:**
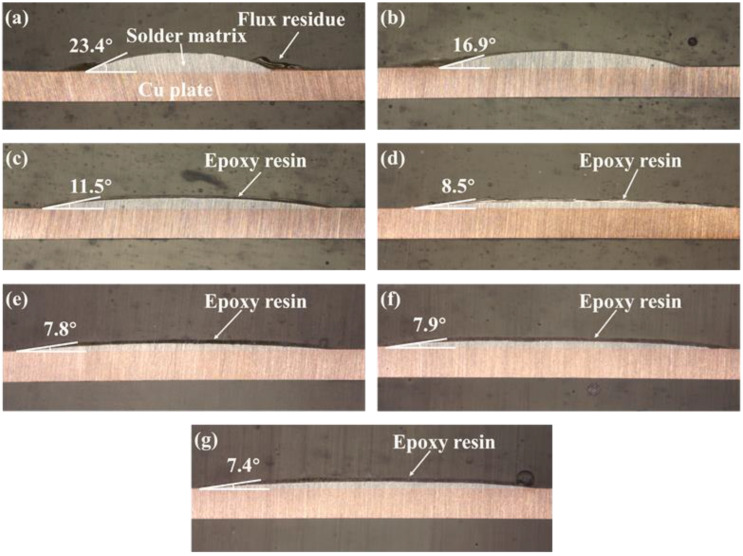
Wetting angles of plain SAC305 and SAC305-xER solder pastes spread on Cu plates: (**a**) plain SAC305, (**b**) x = 2 wt%, (**c**) x = 4 wt%, (**d**) x = 6 wt%, (**e**) x = 8 wt%, (**f**) x = 10 wt%, (**g**) x = 12 wt%.

The surface and cross-sectional morphologies of the spreading specimens of SAC305 and SAC305-*x*ER solder pastes on the Cu plates are shown in Figure 3 and Figure 4, respectively. As shown in Figure 3a and Figure 4a, without resin addition, only the flux residue could be observed at the edge of the spreading sample of the plain SAC305 solder paste. However, when the epoxy resin contents exceeded 4 wt%, complete and uniform resin protective layers could be observed on the surface of spreading samples, as shown in Figure 3c–g and Figure 4c–g.

**Figure 5:**
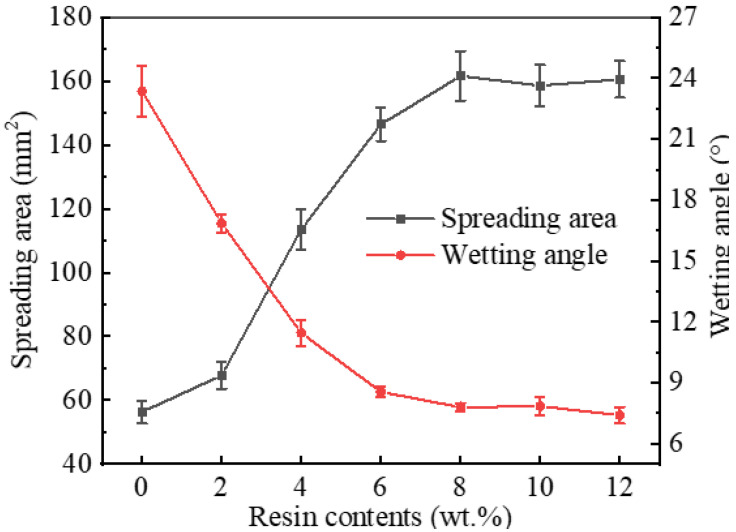
Spreading area and wetting angle of plain SAC305 and SAC305-ER solder pastes on Cu plates.

The spreading area and wetting angle were measured, and their variation trend with epoxy resin content is shown in Figure 5. When the proportion of epoxy resin in the solder pastes was at most 8 wt%, the spreading areas of SAC305-ER composite solder pastes on Cu plates grew rapidly with the increase in epoxy resin content. The spreading area of SAC305-8ER composite solder pastes reached 161.6 mm^2^, which was 187% higher than that of plain SAC305 solder pastes (56.3 mm^2^). Nevertheless, the spreading area remained unchanged with a further increase in resin content. However, the wetting angle showed the opposite variation trend and dropped from 23.4° to 7.4° when 12 wt% epoxy resin was added.

In the spreading test, the weight of the solder paste was kept unchanged for each sample, which means the proportion of solder power decreased when more epoxy resin was added. However, it can be seen that the wettability and spreadability of SAC305 solder pastes improved with the addition of epoxy resin. One reason for this phenomenon is that the acid anhydride curing agent could remove the oxides on the Cu plates while curing the epoxy resin, thereby lowering the interfacial tension of the molten solder/Cu plate. Organic amines, organic acids, and anhydrides are often added into solder pastes as activators to reduce the surface tension of molten solder [23,24]. Another reason is that the epoxy resin system with low viscosity during curing could increase the fluidity of the solder pastes, which promotes the spreading of solder power before melting [25]. 

It should also be noticed that when the resin content is greater than 8 wt%, the spreading performance is hard to enhance further. However, the viscosity of the composite solder pastes may decrease continuously, which will affect the screen-printing process.

### 3.2. Microstructure

**Figure 6:**
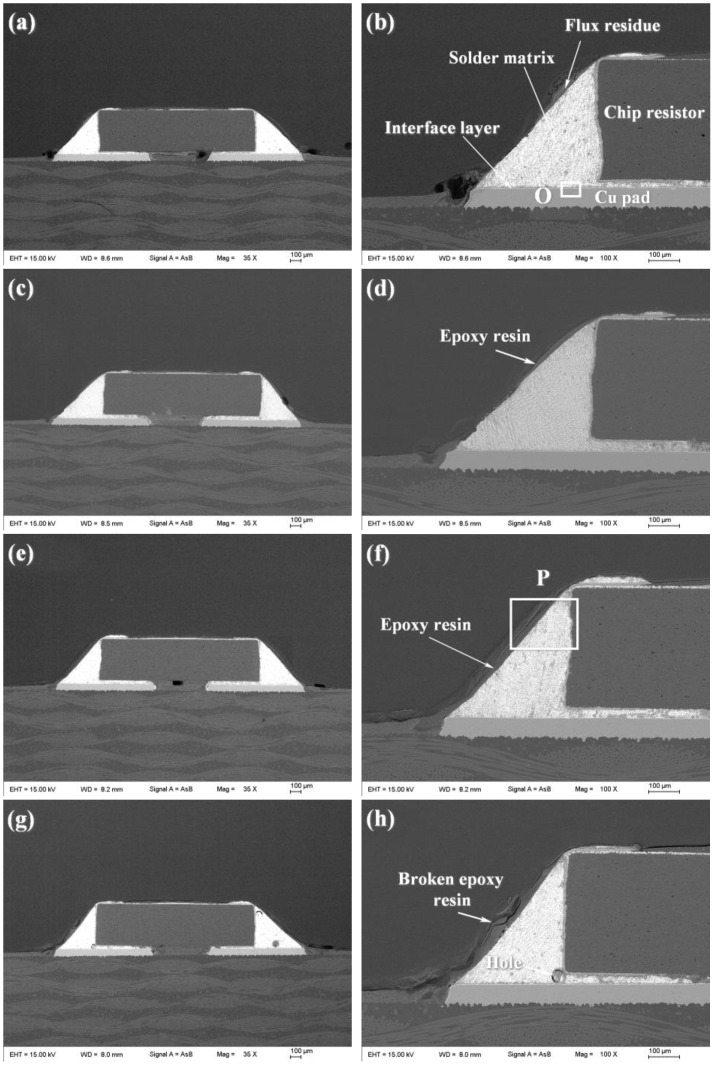
Cross-section SEM images of plain SAC305 and SAC305-ER solder joints: (**a**,**b**) plain SAC305, (**c**,**d**) x = 4 wt%, (**e**,**f**) x = 8 wt%, (**g**,**h**) x = 12 wt%.

**Figure 7:**
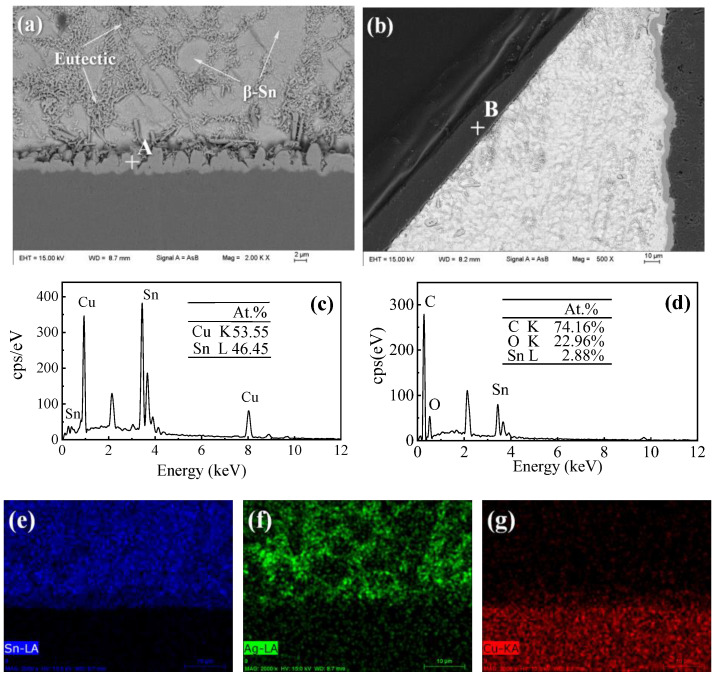
Microstructure analysis of region O and region P in Figure 6: (**a**) SEM image of region O, (**b**) SEM image of region P, (**c**) EDS result of point A, (**d**) EDS result of point B, (**e-g**) EDS elemental mapping results of region O.

The cross-section microstructures of plain SAC305 and SAC305-ER composite solder joints are shown in Figure 6. It can be seen from Figure 6b that the whole solder joint in this study consisted of a chip resistor, solder matrix, interface layer, and Cu pad, in which the solder matrix and interface layer played the central role of electrical and mechanical connection [2]. Figure 7a shows the magnified microstructure of region O in the plain SAC305 solder joints near the interface. The near-eutectic SAC305 solder matrix consisted of β-Sn and eutectic phase, and the interface layer presented a scallop-like shape, which was consistent with previous studies [26,27]. According to the EDS analysis, the interface layer consisted of Cu-Sn IMCs, and the atom ratio of Cu to Sn was about 6:5. Combined with Cu-Sn phase diagram [28] and numerous related studies [29,30,31], the interface IMCs can be determined as Cu_6_Sn_5_ IMCs, which were formed by the reaction between Cu atoms from Cu pad and Sn atoms from the molten solder during reflow soldering. After reflow soldering, some no-clean flux residue remained at the surface of the solder joint.

When 4 wt% epoxy resin was added, a thin epoxy resin layer existed on the surface of the solder matrix, shown in Figure 6d. However, the epoxy resin could not cover the whole surface and presented an uneven morphology. When the resin content reached 8 wt%, an even and complete epoxy resin layer was formed on the joint surface after reflow soldering, as shown in Figure 6f. The formation of the cured epoxy resin layer in the solder joints was similar to that in spreading samples, which was attributed to the simultaneous curing process of the added epoxy that took place during reflow soldering. The magnified image of region P is presented in Figure 7b, and EDS results further confirmed that the protective layer consisted of C-O compounds formed after the epoxy resin was cured.

With the excessive addition of epoxy resin into SAC305-ER composite solder pastes, several holes were found in the as-soldered solder joint, and the epoxy resin layer was not uniform in thickness and exhibited a broken morphology, as shown in Figure 6h. The reason may be that a large amount of added organics could not emerge from the molten solder during the limited reflow time and remained in the solder joints to form holes [16]. Moreover, due to the inherent brittleness of cured epoxy resin, the epoxy resin layer cracked during solidification, which reduced its protection and enhancement effect on the solder joints. For Sn-based solder joints, the solder matrix and the IMC interface layer provide electronic connection and most mechanical support in solder joints, and thus the resin content should be controlled at a rational range to avoid generating such microstructure defects. Therefore, the resin content should be controlled in an appropriate range.

**Figure 8:**
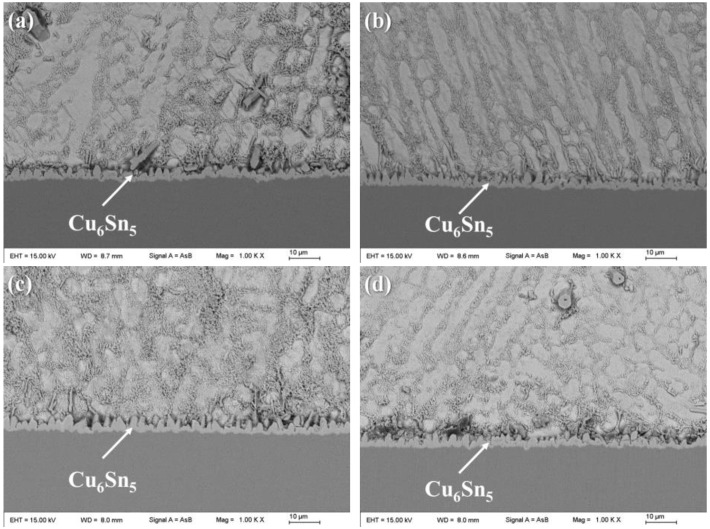
Interfacial morphologies of plain SAC305 and SAC305-ER solder joints: (**a**) plain SAC305, (**b**) x = 4 wt%, (**c**) x = 8 wt%, (**d**) x = 12 wt%.

Figure 8 shows the interfacial morphology of plain SAC305 and SAC305-ER composite solder joints. It could be observed that a continuous and scallop-shape Cu_6_Sn_5_ layer formed between the solder matrix and the Cu pad, regardless of whether the epoxy resin was added into the solder pastes. The interfacial reaction rate was mainly affected by soldering temperature and time, and thus, an interface layer with a thickness of about 3.0 μm was formed in all solder joints due to the same reflow soldering profile.

In summary, the effect of adding epoxy resin on the solder joints’ microstructure is mainly reflected in the formation of the epoxy resin layer, while the phase composition of the solder matrix and the thickness of the interface layer remain almost unchanged.

### 3.3. Shear Force

**Figure 9:**
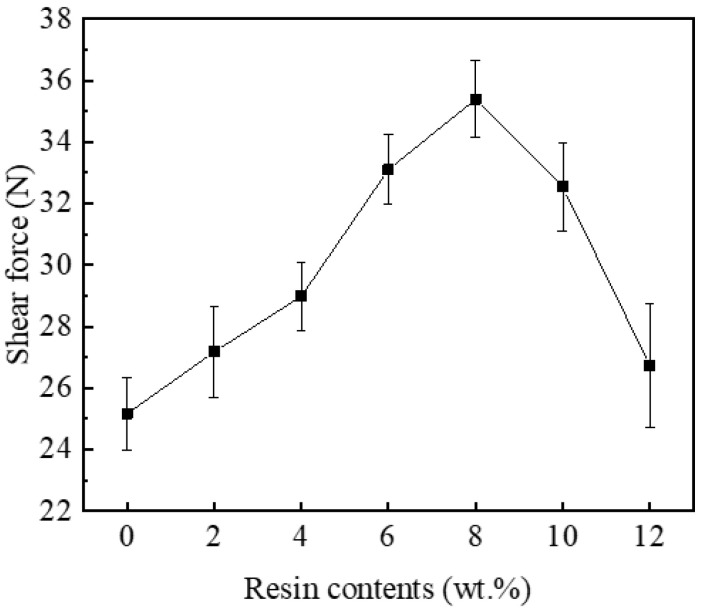
Shear forces of plain SAC305 and SAC305-ER solder joints.

According to the microstructure observation, adding epoxy resin into SAC305 solder pastes changed the macroscopic morphologies of the corresponding joints, which could impact their mechanical properties. In this study, the shear test was conducted to evaluate the role of the cured epoxy resin layers when the solder joints were subjected to shear loads, and the test results are shown in Figure 9. After reflow soldering, a continuous interface layer with suitable thickness was formed in all solder joints, meaning that reliable bonding was built between the solder matrix and Cu substrate. The shear forces of SAC305-ER composite solder joints gradually rose with the increase in resin content and reached the maximum value of 35.39 N with the addition of 8 wt% resin. When excessive resin was added, the shear forces began to decrease. However, the shear force of SAC305-12ER solder joint was 26.73 N, which was still higher than that of the plain SAC305 solder joint (25.17 N).

From the microstructure observation, it can be seen that a complete resin protective layer could not be formed with the addition of 4 wt% epoxy resin. Thus, the improvement of the shear force of SAC305-ER composite solder joints was not noticeable. It benefited from the improved spreadability and wettability of SAC305-4ER composite solder pastes, which promoted the interaction between the molten solder and Cu substrate. When the resin content was further increased, the continuous epoxy resin layer formed during reflow soldering was tightly attached to the solder joint surface. The epoxy resin layer covering the solder joints could share part of the shear stress during the shear test. Thus, the shear performance of the whole composite solder joints was enhanced by the extra mechanical support. However, when the composite solder pastes contained excessive epoxy resin, these organic compounds could not overflow from the molten solder in time during reflow soldering, and remained in the solder matrix to form holes during solidification (as shown in Figure 6g,h), which reduced the uniformity of the solder joints. Meanwhile, the broken epoxy resin layer adhered to the solder joint surface and could not provide sufficient extra mechanical bonding, which weakened the mechanical reinforcement of the epoxy resin layer. Therefore, when the resin content was further increased, the shear force of SAC305-ER composite solder joints gradually reduced, but it was still higher than that of the plain solder joint.

### 3.4. Fracture Morphology

**Figure 10:**
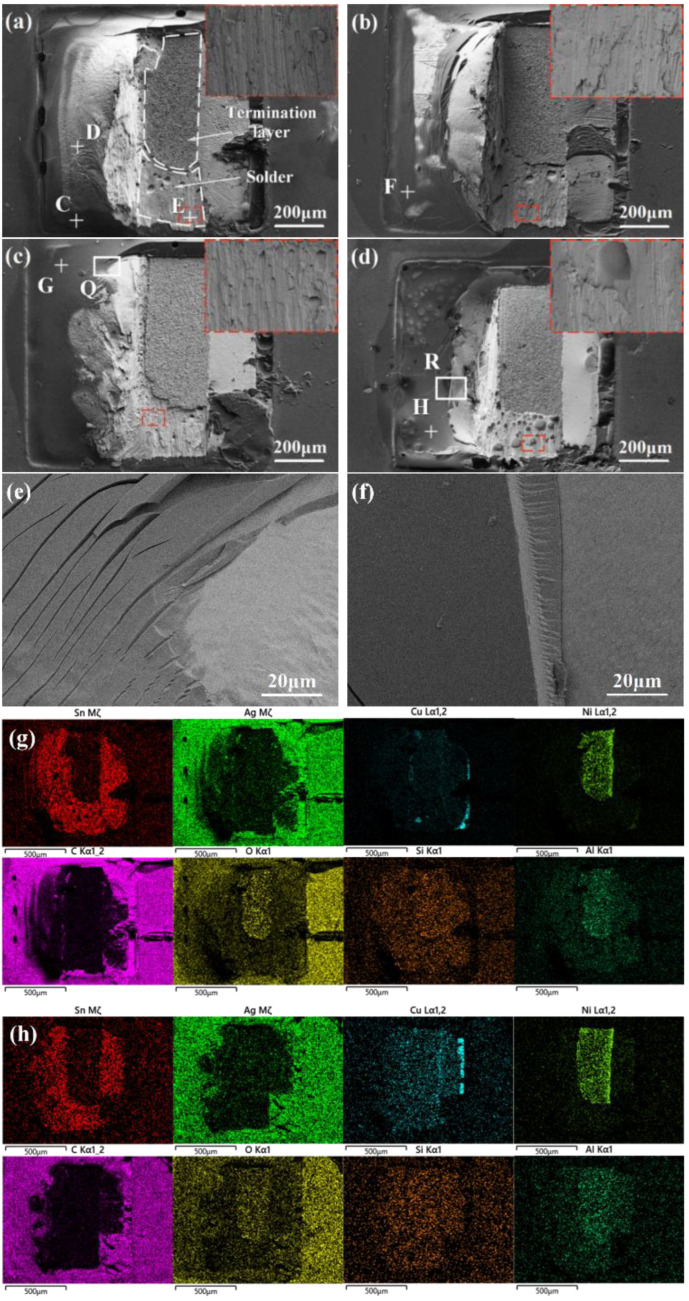
Fracture surfaces of plain SAC305 and SAC305-ER solder joints: (**a**) plain SAC305, (**b**) *x* = 4 wt%, (**c**) *x* = 8 wt%, (**d**) *x* = 12 wt%, (**e**) enlarged image of region Q, (**f**) enlarged image of region R, (**g**) the elemental mapping of (**a**), (**h**) the elemental mapping of (**c**).

The addition of epoxy resin into SAC305 solder pastes brought an obvious mechanical reinforcement of the solder joints, and thus, the fracture morphology analysis was conducted to clarify the epoxy resin layer enhancement during the shear test. Figure 10 presents the fractured surfaces of plain SAC305 and SAC305-ER composite solder joints after the shear test. The EDS mapping analysis indicated that regardless of whether epoxy resin was added to the solder paste, the fracture position of all the solder joints was partially in the solder and partially in the termination layer of the chip resistor.

It can be seen from Figure 10a and Table 1 that some flux residue (such as point C) remained at the fracture surface of the SAC305 solder joint, which could hardly provide mechanical support for the solder joint under shear stress. With the addition of 4 wt% epoxy resin, discontinuous cured epoxy resin (such as point F) could be observed at the partial surface of the fractured joint. The coverage of epoxy resin was small due to the low proportion, and thus, the enhancement of epoxy resin on the shear property of the solder joint was limited.

During reflow soldering, the cross-linking reaction occurred between the epoxy resin and the curing agent to form a cured product. When 8 wt% epoxy resin was added into SAC305 solder pastes, most of the solder joint surface was covered by an epoxy resin layer. The epoxy resin layer that adhered to the surface also undertook part of the shear stress during the shear test, and thus, the shear force was improved significantly. The deformation and fracture of the epoxy resin layer can be observed in the magnified SEM image of region Q in Figure 10e. With the excessive addition of 12 wt% epoxy resin, it could be seen that the thickness of the adhered resin layer was further increased and the resin was also broken after the shear test (as shown in Figure 10f).

The addition amount of epoxy resin into the solder paste determined the morphology and thickness of epoxy resin layer on the solder joint surface. However, the fracture modes of the joints bearing different epoxy resin contents did not change. As shown in the magnified fracture surface images (red insets) in the upper right corner of Figure 10a–d, obvious shear traces existed on the fracture morphologies and presented ductile fracture characteristics. However, there were many holes on the fracture of the SAC305-12ER composite solder joint (as shown in Figure 10d); the reason may be that the excessive epoxy resin added could not float out of the molten solder in time during reflow soldering, and remained in the solder to form holes after solidification. Considering that the generation of a large number of holes will destroy the structural integrity of the solder joint, the proportion of added epoxy resin should be controlled at about 8 wt%.

## 4. Conclusions

In this paper, the influence of adding epoxy resin on the wettability, spreadability, and shear behaviour of SAC305 solder pastes was investigated, and the following conclusions can be drawn:

1. The addition of epoxy resin effectively increased the fluidity of SAC305 solder pastes and reduced the surface tension of the molten solder, and thus, the spreading performance and wettability of SAC305 solder pastes were improved.
2. When epoxy resin content reached 8 wt%, a continuous and complete epoxy resin layer was formed on the as-reflowed joint surface. However, the broken epoxy resin layer and several holes were found in the composite solder joint bearing excessive epoxy resin. Regardless of the added amount of epoxy resin, the morphology and thickness of the interfacial layer hardly changed.
3. Due to the mechanical blocking effect of the epoxy resin layer, the shear force of the SAC305 solder joint enhanced by epoxy resin significantly increased and reached a maximum value of 35.39 N when the epoxy resin content was 8 wt%. Fracture analysis showed that the fracture mode of all solder joints was ductile fracture, but the deformation and fracture of the resin layer on the surface of the composite solder joints containing 8 wt% or more epoxy resin were observed, indicating that the epoxy resin layer also shared part of the stress in the shear test.

## Figures and Tables

**Table 1:** EDS results of different points in Figure 10.

Points	Composition (at %)				
	C	O	Sn	Ag	Cu
C	89.34	10.66	-	-	-
D	-	-	93.82	5.41	0.77
E	-	-	99.24	0.00	0.76
F	83.99	16.01	-	-	-
G	83.03	16.97	-	-	-
H	82.19	17.81	-	-	-

## Data Availability

Not applicable.
